# Progressive Rochdale

**Published:** 1924-12

**Authors:** 


### PROGRESSIVE ROCHDALE.

Rochdale has lately had a particularly "live" Health Week. The Committee brought its propaganda before the townspeople by inviting preachers in churches and chapels to address their congregations, adult classes and men's meetings on Health, by arranging that short talks on the subject should be given to elementary scholars, and that special Health films should be shown at the local cinemas, so that, whether they wanted it or not, the town's inhabitants simply could not get away from health matters. Public interest having been thus roused, there was a free Health Exhibition to be seen all the week at the Art Gallery, where exhibits were shown mainly supplied by the Central Council for Infant and Child Welfare. There were lectures in the Town Hall by such authorities as Dr. Saleeby, Dr. Eric Pritchard, Dr. J. T. Ewart and Dr. Allen Daley, who, in accordance with Rochdale's method of going to the root of a matter, lectured on "The Beginnings of Disease." We feel sure that so well-planned and comprehensive a Health Week is bound to have widely beneficial results.

### A CHESTER TRANSFORMATION.

The recent opening of the Humberston wing at Chester Royal Infirmary completes a policy begun in 1912. It arose from the bequest and gifts of the late Col. Humberston, whose last and greatest services to the city were performed during the twenty years when he was chairman of the Infirmary. The present chairman, Mr. E. Sneyd Kynnersley, has recorded that twelve years ago, so far as he could discover, there were only seven nurses' bedrooms, to be replaced by a proper home in the opening year of the war. The Humberston wing, of which Mr. Campbell is the architect, provides among other accommodation a further twenty bedrooms. Paying patients are now being arranged for. All this has been made possible by the gift mentioned, together with the late Mr. Boden's legacy of nearly £5,000, so that the cost of the new wing has not been borne by the hospital's funds, nor has it been opened with any debt. This is lucky, for £25,000 is needed to pay off arrears on the general account before the middle of 1925. The pathological department, a recent improvement, is proving of great use to Chester and indeed to two other counties also. Those who remember the Infirmary as it was before 1912 will realise that it has been transformed in the interval.

